# Effect of continuity of patient care on quality of life and psychological state in patients with inflammatory bowel disease: a systematic review and meta-analysis of randomized controlled trials

**DOI:** 10.7717/peerj.21429

**Published:** 2026-07-14

**Authors:** Xiu-Rong Huang, Hui Duan, Xin-Xu Zhou, Min Wang, Jin Wang

**Affiliations:** 1 Department of Rheumatology and Immunology, The Chengdu Third People’s Hospital, Chengdu, Sichuan, China; 2 Department of Gastroenterology, The Chengdu Third People’s Hospital, Chengdu, Sichuan, China

**Keywords:** Inflammatory bowel disease, Crohn disease, Colitis ulcerative, Continuity of patient care, Quality of life, Anxiety, Depression, Systematic review, Meta-analysis

## Abstract

**Purpose:**

To systematically evaluate the effectiveness of continuity of patient care on quality of life, anxiety, and depressive symptoms in inflammatory bowel disease.

**Methods:**

Five databases were systematically searched for relevant studies from inception to March 20, 2026. The primary outcome measure included quality of life, anxiety, and depression state. The tools of Cochrane’s risk-of-bias were used to assess bias risks. Then, the fixed effect or the random effect model was applied in the meta-analysis. Continuity variables were expressed as standardized mean difference (SMD) and its 95% confidence interval (95% CI), while dichotomous variables were presented as relative risk (RR) and its 95% CI. Finally, sensitivity and publication bias were performed.

**Results:**

Twelve randomized controlled trials involving 1,246 participants were included. Continuity of patient care significantly improved quality of life (including inflammatory bowel disease questionnaire and 36-item Short Form Health Status Survey), as well as the anxiety (SMD = −1.98, 95% CI [−2.40 to −1.55], *p* < 0.00001) and depression (SMD = −1.82, 95% CI [−2.67 to −0.98], *p* < 0.0001) levels of IBD patients. They did not significantly reduce the recurrence rate (SMD = 0.94, 95% CI [0.53–1.67], *p* = 0.83) and nursing satisfaction (SMD = 1.19, 95% CI [0.97–1.45], *p* = 0.10).

**Conclusions:**

This meta-analysis revealed that continuity of patient care might improve the quality of life and anxiety and depression levels of patients with inflammatory bowel disease but not the recurrence rate. This meta-analysis provides novel insights into continuity of patient care in the field of IBD. Healthcare workers should practice continuity in patient care for patients with inflammatory bowel disease to improve the quality of life and anxiety and depression. Due to extremely high heterogeneity, we lowered the evidence to a moderate quality level.

## Introduction

As is well documented, inflammatory bowel disease (IBD), encompassing Crohn’s disease and ulcerative colitis, is a chronic and idiopathic gastrointestinal disease. IBD, closely related to Western dietary culture, is more prevalent in Western countries (Ananthakrishnan, 2015). At present, one million patients in the United States and 2.5 million patients in Europe suffer from IBD (Ananthakrishnan, 2015). With improving living standards and the integration of Chinese and Western dietetic cultures, the incidence of IBD in Asian populations is on the rise (Ran et al., 2020). IBD generally manifests as chronic abdominal pain, intestinal obstruction, diarrhea, pus, blood stools, fever, and mucus, with patients experiencing unbearable pain due to repeated relapse (Baumgart & Sandborn, 2012). Additionally, according to BE-FIT-IBD studies (Gravina et al., 2023), the reduced propensity to engage in physical activity was observed in IBD patients. Due to its recurrent, remittent, and incurable nature, the quality of life (QoL) of patients is severely affected (Litta et al., 2019). As defined by the World Health Organization (WHO), QoL is an individual’s perception of their place in life within the context of their cultures and value systems and in relation to their goals, expectations, and aspirations (Coyne et al., 2017). Its four dimensions most commonly considered during assessment include motor activity, functional aspects, psychological aspects, and social aspects (Janz et al., 2013). The physical symptoms of IBD also contribute to chronic fatigue and depression, which are associated with a lower QoL (Mitchell et al., 1988; Van Langenberg & Gibson, 2010; Jelsness-Jørgensen et al., 2010; Kang et al., 2022). In addition, patients with IBD face a much greater risk of anxiety and depression compared to the general population (Choi et al., 2019; Ryu et al., 2022). The severity of the disease, not following treatment plans, and socioeconomic challenges are factors connected to heightened anxiety and depression (Nahon et al., 2012). Furthermore, disability associated with IBD has a detrimental impact on quality of life (Keller et al., 2020). Numerous studies (Alvestad et al., 2022; Ramos et al., 2023; Aguas Peris et al., 2024; Sheehan et al., 2024; Yu et al., 2025) over the past five years have reported the application of continuity of patient care interventions in IBD. Continuity of care is an essential principle in healthcare, notably in nursing and primary care. A commonly cited definition by Haggerty et al. (2003) describes continuity as: a patient’s and their care team’s view of a well-organized and uninterrupted care journey over time. This definition is based on three connected aspects: (1) Informational continuity involves using data from previous events and personal situations to tailor current care for each person. (2) Management continuity involves delivering consistent and coordinated care across various providers and settings. (3) Relational continuity involves maintaining a continuous therapeutic relationship between a patient and their healthcare providers. Additionally, continuity of care stresses the importance of holistic evaluations, discharge planning centered on the patient, transitional care actions, and collaboration among different professions to ensure smooth transitions for patients without any gaps. The concept of continuity of patient care is about bringing high-quality healthcare services into the home to monitor patients’ compliance, treatment outcomes, and psychological well-being post-discharge (Brooten et al., 2002; Li, Zhou & Lin, 2020). Nonetheless, the intervention effects of continuity of patient care remain controversial (Borgaonkar et al., 2002; Abutaleb et al., 2018). Thus, there is a pressing need to conduct a comprehensive systematic review and meta-analysis focusing on the continuity of patient care. This study’s research objectives is to systematically evaluate the effectiveness of continuity of patient care on quality of life, anxiety, and depressive symptoms in inflammatory bowel disease.

## Methods

This meta-analysis was performed in accordance with the Cochrane Handbook for Systematic Reviews of Interventions and complied with the Preferred Reporting Items for Systematic Reviews and Meta-Analyses (PRISMA) guideline (Arya, Kaji & Boermeester, 2021; Higgins et al., 2024).

### Data source and searches

Two authors independently searched PubMed, Web of Science, Cochrane Library, Chinese National Knowledge Infrastructure (CNKI), and WanFang Data Knowledge Service Platform (WanFang) to screen relevant studies. During the literature search, no language restrictions were imposed, and papers were retrieved until March 20, 2026. The search results were then screened against pre-defined inclusion criteria. Due to resource constraints for translation, only studies published in English or Chinese were included in the final analysis. To prevent omissions, a search method combining Mesh terms and free words was employed. Additionally, manual searches were performed based on cited references. The PubMed search strategy was as follows:

#1 Inflammatory Bowel Diseases [MH] OR Crohn Disease [MH] OR Colitis, Ulcerative [MH] OR Inflammatory Bowel Diseases OR Inflammatory Bowel Disease OR Bowel Diseases, Inflammatory OR Crohn Disease OR Crohn’s Enteritis OR Regional Enteritis OR Crohn’s Disease OR Crohns Disease OR Inflammatory Bowel Disease 1 OR Enteritis, Granulomatous OR Granulomatous Enteritis OR Enteritis, Regional OR Ileocolitis OR Colitis, Granulomatous OR Granulomatous Colitis OR Ileitis, Terminal OR Terminal Ileitis OR Ileitis, Regional OR Regional Ileitide OR Regional Ileitis OR Colitis, Ulcerative OR Idiopathic Proctocolitis OR Ulcerative Colitis OR Colitis Gravis OR Inflammatory Bowel Disease, Ulcerative Colitis Type

#2 Continuity of Patient Care [MH] OR Continuity of Patient Care OR Care Continuity, Patient OR Patient Care Continuity OR Continuum of Care OR Care Continuum OR Continuity of Care OR Care Continuity

#3 randomized controlled trial [PT] OR controlled clinical trial [PT] OR randomized OR randomly OR trial OR groups

### Inclusion and exclusion criteria

#### Inclusion criteria

The inclusion criteria were based on the PICOS approach as follows:

1. Participant (P): Adults diagnosed with IBD

2. Intervention (I): Continuity of patient care initiated during in-patient hospitalization or within two weeks after discharge.

3. Comparison (C): Any form of non-continuity of patient care except continuity of patient care, such as conventional care, standard care, usual care, *etc.*

4. Outcomes (O)

(I) Primary outcomes: QoL was evaluated using the inflammatory bowel disease questionnaire (IBDQ) (Chen et al., 2016; Huang, 2017; Sun et al., 2021; Yu, Ren & Yao, 2022) and 36-item Short Form Health Status Survey (SF-36) (Elkjaer et al., 2010; Peng et al., 2015; Ma et al., 2019). The psychological state of patients was evaluated using the self-rating anxiety scale (SAS) (Ma et al., 2019; Meng, Cui & Wang, 2020; Zhang et al., 2025) and self-rating depression scale (SDS) (Ma et al., 2019; Meng, Cui & Wang, 2020; Zhang et al., 2025).

(II) Secondary outcome: disease recurrence, medication adherence, medical advice compliance, and nursing satisfaction.

5. Study design (S): Only randomized controlled trials (RCTs) published in English or Chinese.

#### Exclusion criteria

The exclusion criteria were as follows:

1. Literature reviews, dissertations, commentaries, conference abstracts, editorials, and study protocols.

2. Studies without available full-text.

3. Studies where patients in each group received continuity of patient care.

4. Studies that did not provide outcomes of interest.

### Study selection and data extraction

Two investigators independently screened the literature for eligibility, and any potential conflicts were resolved through discussion or consultation with a third investigator. First, the retrieved citations were imported into EndNote X9 software. Then, the titles were manually reviewed to exclude duplicates. Secondly, the title and abstract of each article were screened according to the inclusion and exclusion criteria. Studies were initially considered only if published in journals indexed in Chinese Scientific and Technical Papers and Citation Database (CSTPCD) or Chinese Science Citation Database (CSCD). This criterion was applied to ensure a baseline level of methodological quality. After excluding low-quality literature (None CSTPCD, the CSCD), the full text of the retained articles was reviewed against the predefined inclusion and exclusion criteria. To ensure the sufficiently comparability of included interventions, only studies which clearly used the “Continuity of patient care” were included in this systematic review and meta-analysis. Lastly, data were independently extracted by two authors, and disagreements were resolved by arbitration by a third investigator. The extracted data comprised authors, publish time, country, IBD type, sample size, male/female ratio, age, course, intervention or follow-up time, and outcomes.

### Quality assessment

Studies included in this review were independently evaluated by two investigators according to the Cochrane risk of bias tool, which assesses random sequence generation (selection bias), allocation concealment (selection bias), blinding of participants and personnel (performance bias), blinding of outcome assessment (detection bias), incomplete outcome data (attrition bias), selective reporting (reporting bias), and other biases (Higgins & Green, 2019). Each item was categorized as “low risk”, “high risk”, and “unclear risk” of bias. Disagreements were resolved through discussion or consultation with a third investigator.

### Statistical analysis

The meta-analysis was conducted using the EndNote X9 and Review Manager 5.4.1. Continuity variables were expressed as standardized mean difference (SMD) and its 95% confidence interval (95% CI), whereas dichotomous variables were presented as relative risk (RR) and its 95% CI. Heterogeneity was assessed using the I^2^ test, with values exceeding 50% indicating significant heterogeneity between studies (Knoll et al., 2017). The fixed effects model was adopted when I^2^ values ≤50%; otherwise, the random effects model was applied. Sensitivity analysis was performed to explore sources of heterogeneity by systematically excluding each study and monitoring its impact on the overall effect size. In cases where more than 10 studies were included, publication bias was assessed using a funnel plot (Sterne et al., 2011; Chen et al., 2023).

## Results

### Search results and selection

**Figure 1:**
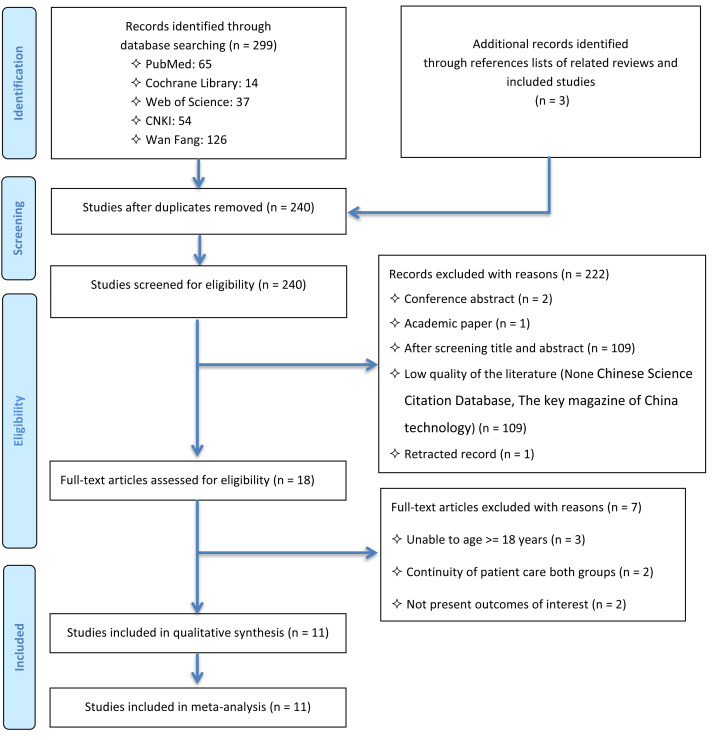
PRISMA diagram showing selection of articles for review.

In total of 299 articles were initially identified from three English databases and two Chinese databases according to a predesigned literature retrieval strategy. An additional three articles were identified *via* manual search. One article by Elkjaer et al. (2010) included two separate RCTs in Denmark and Ireland and was thus treated as two independent studies. Ultimately, 12 studies (Elkjaer et al., 2010; Peng et al., 2015; Chen et al., 2016; Huang, 2017; Wu, Fu & Kang, 2018; Ma et al., 2019; Meng, Cui & Wang, 2020; Zhou, 2020; Sun et al., 2021; Yu, Ren & Yao, 2022; Zhang et al., 2025) were included in the systematic review and meta-analysis according to inclusion and exclusion criteria. Figure 1 illustrates the screening and exclusion process in a flow diagram.

### Study characteristics

A total of 1,246 IBD patients were included in this meta-analysis, including 661 male patients and 585 female patients. Their mean ranged between 25.28 and 68.1 years. The majority of retrieved articles were published in China and were single-center studies. They were published between 2010 and 2025 and were conducted in China (*n* = 10) (Peng et al., 2015; Chen et al., 2016; Huang, 2017; Wu, Fu & Kang, 2018; Ma et al., 2019; Meng, Cui & Wang, 2020; Zhou, 2020; Sun et al., 2021; Yu, Ren & Yao, 2022; Zhang et al., 2025), Denmark, and Ireland (*n* = 2) (Elkjaer et al., 2010). The intervention or follow-up ranged between two months (Ma et al., 2019) and 12 months (Elkjaer et al., 2010; Zhou, 2020). Table 1 showed the characteristics of included studies.

### Quality assessment

**Figure 2:**
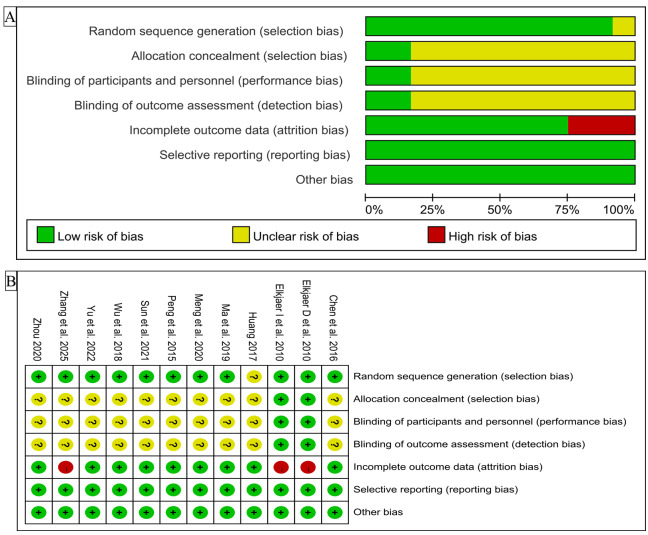
Risk of bias graph.

Figure 2 displays the risk of bias for all included studies. One study (Huang, 2017) did not report the generation of random sequences and were rated as unclear risk. Only two studies (Elkjaer et al., 2010) reported adequate allocation concealment and blinding of participants and personnel and were rated as low risk. The remaining studies (Peng et al., 2015; Chen et al., 2016; Huang, 2017; Wu, Fu & Kang, 2018; Ma et al., 2019; Meng, Cui & Wang, 2020; Zhou, 2020; Sun et al., 2021; Yu, Ren & Yao, 2022; Zhang et al., 2025) were rated as unclear risk. Three studies (Elkjaer et al., 2010; Zhang et al., 2025) reported incomplete outcome data and were rated as high risk. All studies (Peng et al., 2015; Chen et al., 2016; Elkjaer et al., 2010; Huang, 2017; Wu, Fu & Kang, 2018; Ma et al., 2019; Meng, Cui & Wang, 2020; Zhou, 2020; Sun et al., 2021; Yu, Ren & Yao, 2022; Zhang et al., 2025) showed no evidence of reporting bias or other biases.

**Table 1:** Characteristics of included studies.

Authors (Year)	Country	IBD type	Study design	Sample size (n)		Male/female (n)		Age (Year)		Course (Year, Months)		Intervention/ follow-up time	Outcomes
				Observation	Control	Observation	Control	Observation	Control	Observation	Control		
Yu, Ren & Yao (2022)	China	IBD	RCT; Single-centre	85	85	45/40	48/37	44 ± 1	46 ± 2	> 0.5	> 0.5	3 Months	①
Sun et al. (2021)	China	UC/CD	RCT; Single-centre	41	41	23/18	27/14	31.05 ± 11.01	31.78 ± 11.32	3	3	6 Months	①⑧
Zhou (2020)	China	IBD	RCT; Single-centre	40	40	21/19	23/17	40.83 ± 5.03	40.57 ± 4.96	16.48 ± 4.36 M	17.40 ± 4.58 M	12 Months	①
Meng, Cui & Wang (2020)	China	UC	RCT; Single-centre	48	48	27/21	28/20	38.21 ± 4.44	38.62 ± 4.64	4.39 ± 1.23	4.28 ± 1.26	3 Months	③④
Ma et al. (2019)	China	UC	RCT; Single-centre	46	46	27/19	21/25	41.94 ± 4.78	40.85 ± 5.19	5.01 ± 2.36	5.26 ± 2.13	2 Months	①②③④⑤
Wu, Fu & Kang (2018)	China	UC	RCT; Single-centre	44	44	24/20	26/18	41.30 ± 8.64	40.61 ± 7.98	5.36 ± 1.20	5.41 ± 1.52	6 Months	①⑥
Huang (2017)	China	UC	RCT; Single-centre	32	32	14/18	15/17	51.6 ± 7.4	49.3 ± 6.8	NA	NA	6 Months	①
Chen et al. (2016)	China	UC	RCT; Single-centre	40	40	26/14	28/12	68.1 ± 3.5	67.8 ± 3.0	5.4 ± 1.7	5.0 ± 1.5	NA	①⑥⑧
Peng et al. (2015)	China	UC	RCT; Single-centre	30	30	17/13	11/19	53.75 ± 12.1	54.34 ± 11.4	5.38 ± 1.79 M	5.46 ± 1.53 M	3 Months	②
Zhang et al. (2025)	China	IBD	RCT; Single-centre	50	51	34/16	32/19	25.28 ± 3.17	25.42 ± 3.59	NA	NA	6 Months	③④
Elkjaer et al. (2010)	Denmark	UC	RCT; Multi-centre	117	116	57/60	35/81	33	35	4	6	12 Months	⑤⑥⑦
Elkjaer et al. (2010)	Ireland	UC	RCT; Multi-centre	52	48	32/20	20/28	30	34	8	8	12 Months	⑤⑥⑦

**Notes.**

Abbreviations IBD inflammatory bowel disease UC ulcerative uolitis CD crohn disease RCT randomized controlled trials M month NA not applicable

Outcomes ①IBDQ inflammatory bowel disease questionnaire ②SF-36 36-Item Short Form Health Status Survey ③SAS Self-rating anxiety scale ④SDS Self-rating depression scale ⑤ disease recurrence rate ⑥ medication adherence rate ⑦ medical advice compliance rate ⑧ nursing satisfaction

### Primary outcomes

#### IBDQ

##### IBDQ total score.

**Figure 3:**
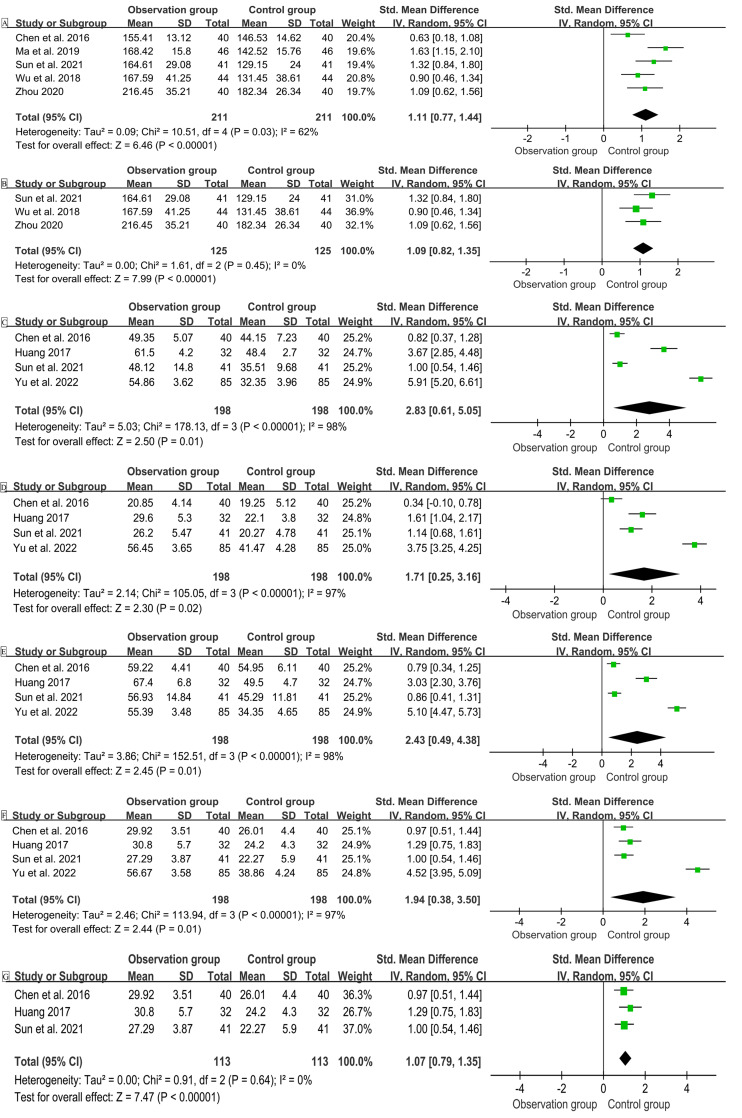
(A) Effects of on IBDQ total score. (B) Sensitivity analysis on IBDQ total score. (C) Effects of on bowel symptoms. (D) Effects of on systemic symptoms. (E) Effects of on emotional function. (F) Effects of on social functioning. (G) Sensitivity analysis on social functioning. Observation group (Intervention): underwent continuity of patient care interventions; Control group: no continuity of patient care interventions.

Five RCTs involving 422 patients (Chen et al., 2016; Wu, Fu & Kang, 2018; Ma et al., 2019; Zhou, 2020; Sun et al., 2021) reported the IBDQ total score. Compared with the conventional care group, the result of a random-effects model revealed that continuity of patient care significantly improved the life quality of patients (SMD = 1.11, 95% CI [0.77–1.44], *p* < 0.00001; Fig. 3A). Sensitivity analysis revealed that studies undertaken by Chen et al. (2016); Ma et al. (2019) were the primary sources of heterogeneity. Their exclusion significantly decreased heterogeneity (SMD = 1.09, 95% CI [0.82–1.35], *p* < 0.00001; Fig. 3B).

##### Bowel symptoms.

Four RCTs involving 396 patients (Sun et al., 2021; Chen et al., 2016; Huang, 2017; Yu, Ren & Yao, 2022) reported bowel symptoms. The random-effects model indicated that continuity of patient care significantly improved bowel symptoms compared with the conventional care group (SMD = 2.83, 95% CI [0.61–5.05], *p* = 0.01; Fig. 3C). Sensitivity analysis demonstrated that the result was unaffected by excluding any single RCTs.

##### Systemic symptoms.

Four RCTs (Sun et al., 2021; Chen et al., 2016; Huang, 2017; Yu, Ren & Yao, 2022) provided data on the systemic symptoms of 396 IBD patients, with Fig. 3D depicting a forest plot of the results, where the random-effects model illustrated continuity of patient care significantly alleviated systemic symptoms in IBD patients (SMD = 1.71, 95% CI [0.25–3.16], *p* = 0.02; Fig. 3D). The meta-analysis result was unaffected by removing any single RCTs, as determined by sensitivity analysis.

##### Emotional function.

Four RCTs (Sun et al., 2021; Chen et al., 2016; Huang, 2017; Yu, Ren & Yao, 2022) involving 396 IBD patients examined emotional function, and pooled results suggested that continuity of patient care was associated with superior emotional function (SMD = 2.43, 95% CI [0.49–4.38], *p* = 0.01; Fig. 3E). The meta-analysis result was unaffected by excluding any single RCTs using the leave-one-out approach, as determined by sensitivity analysis.

##### Social functioning.

Four RCTs (Sun et al., 2021; Chen et al., 2016; Huang, 2017; Yu, Ren & Yao, 2022) involving 396 IBD patients assessed social functioning, and the pooled results exposed that continuity of patient care was associated with superior social functioning (SMD = 1.94, 95% CI [0.38–3.50], *p* = 0.01; Fig. 3F). Sensitivity analysis revealed that studies undertaken by Yu, Ren & Yao (2022) was the primary sources of heterogeneity. Their exclusion significantly decreased heterogeneity (SMD = 1.07, 95% CI [0.79–1.35], *p* < 0.00001; Fig. 3G).

#### SF-36

##### Physical functioning.

**Figure 4:**
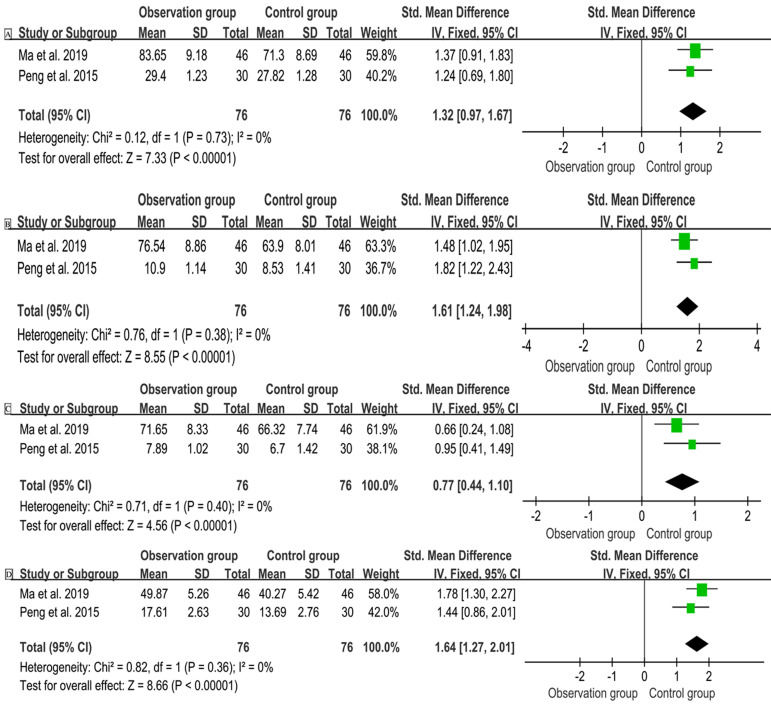
(A) Effects of on SF-36 physical functioning. (B) Effects of on SF-36 bodily pain. (C) Effects of on SF-36 role physical. (D) Effects of on SF-36 general health. Observation group (Intervention): underwent continuity of patient care interventions; Control group: no continuity of patient care interventions.

Two RCTs (Peng et al., 2015; Ma et al., 2019) involving 152 IBD patients evaluated physical functioning. The fixed effects model presented that continuity of patient care significantly enhanced physical functioning compared with the conventional care group (SMD = 1.32, 95% CI [0.97–1.67], *p* < 0.00001; Fig. 4A).

##### Bodily pain.

Two RCTs (Peng et al., 2015; Ma et al., 2019) involving 152 IBD patients investigated bodily pain. The fixed effects model demonstrated continuity of patient care that significantly improved bodily pain compared with the conventional care group (SMD = 1.61, 95% CI [1.24–1.98], *p* < 0.00001; Fig. 4B).

##### Role physical.

Two RCTs (Peng et al., 2015; Ma et al., 2019) involving 152 IBD patients assessed role physical. The meta-analysis demonstrated that the continuity of patient care group was significantly superior to the conventional care group in improving the role physical of IBD patients (SMD = 0.77, 95% CI [0.44–1.10], *p* < 0.00001; Fig. 4C).

##### General health.

Two RCTs (Peng et al., 2015; Ma et al., 2019) involving 152 IBD patients analyzed general health. The meta-analysis demonstrated that the continuity of patient care group was significantly superior to the conventional care group in improving the general health of IBD patients (SMD = 1.64, 95% CI [1.27–2.01], *p* < 0.00001; Fig. 4D).

##### Vitality.

**Figure 5:**
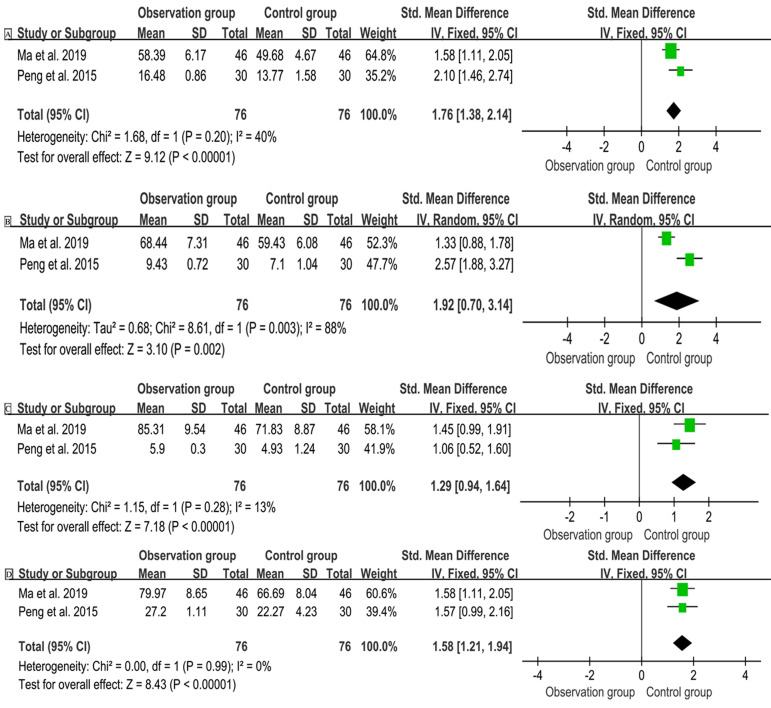
(A) Effects of on SF-36 vitality. (B) Effects of on SF-36 social functioning. (C) Effects of on SF-36 emotional health. (D) Effects of on SF-36 mental health. Observation group (Intervention): underwent continuity of patient care interventions; Control group: no continuity of patient care interventions.

Two RCTs (Peng et al., 2015; Ma et al., 2019) involving 152 IBD patients assessed vitality, and the pooled results showed that continuity of patient care was associated with significantly improved vitality compared to conventional care (SMD = 1.76, 95% CI [1.38–2.14], *p* < 0.00001; Fig. 5A).

##### Social functioning.

Two RCTs (Peng et al., 2015; Ma et al., 2019) involving 152 IBD patients investigated social functioning, and the pooled results unveiled that continuity of patient care was associated with superior social functioning compared to conventional care (SMD = 1.92, 95% CI [0.70–3.14] *p* = 0.002; Fig. 5B).

##### Emotional health.

Two RCTs (Peng et al., 2015; Ma et al., 2019) involving 152 IBD patients analyzed emotional health, and the fixed effects model showed that continuity of patient care was associated with superior role emotional health compared to conventional care (SMD = 1.29, 95% CI [0.94–1.64], *p* < 0.00001; Fig. 5C).

##### Mental health.

Two RCTs (Peng et al., 2015; Ma et al., 2019) involving 152 IBD patients explored mental health. The meta-analysis demonstrated that the continuity of patient care group was significantly superior to the conventional care in improving the mental health of IBD patients (SMD = 1.58, 95% CI [1.21–1.94], *p* < 0.00001; Fig. 5D).

##### Anxiety levels.

**Figure 6:**
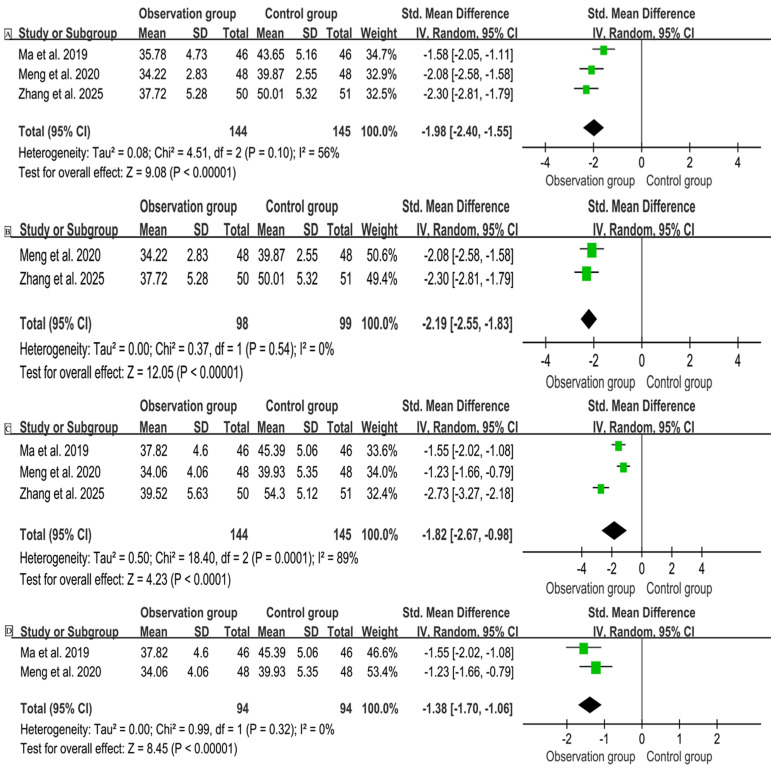
(A) Effects of on SAS. (B) Sensitivity analysis on SAS. (C) Effects of on SDS. (D) Sensitivity analysis on SDS. Observation group (Intervention): underwent continuity of patient care interventions; Control group: no continuity of patient care interventions).

Three RCTs with 289 IBD patients (Ma et al., 2019; Meng, Cui & Wang, 2020; Zhang et al., 2025) reported anxiety levels. The results of the random-effects model showed that continuity of patient care significantly improved the anxiety levels of patients compared with conventional care (SMD = −1.98, 95% CI [−2.40 to −1.55], *p* < 0.00001; Fig. 6A). Sensitivity analysis revealed that studies undertaken by Ma et al. (2019) was the primary sources of heterogeneity. Their exclusion significantly decreased heterogeneity (SMD = −2.19, 95% CI [−2.55 to −1.83], *p* < 0.00001; Fig. 6B).

##### Depression.

Three RCTs involving 289 IBD patients (Ma et al., 2019; Meng, Cui & Wang, 2020; Zhang et al., 2025) reported data on depression. As anticipated, the result of the random-effects model showed that continuity of patient care significantly alleviated depressive symptoms compared to conventional care in IBD patients (SMD = −1.82, 95% CI [−2.67 to −0.98], *p* < 0.0001; Fig. 6C). Sensitivity analysis revealed that studies undertaken by Zhang et al. (2025) was the primary sources of heterogeneity. Their exclusion significantly decreased heterogeneity (SMD = −1.38, 95% CI [−1.70 to −1.06], *p* < 0.00001; Fig. 6D).

### Secondary outcome

#### Disease recurrence rate

**Figure 7:**
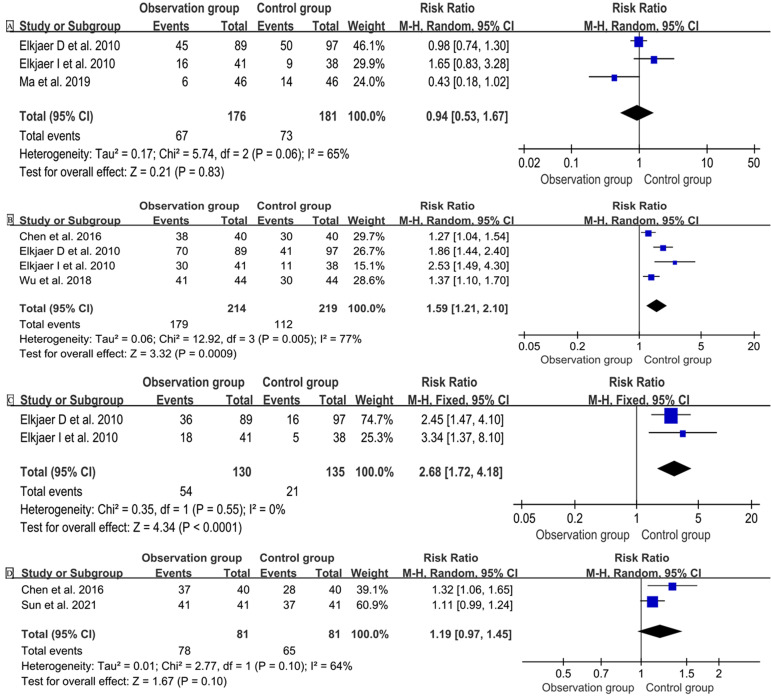
Effects of continuity of patient care interventions on (A) disease recurrence rate, (B) medication adherence rate, (C) medical advice compliance, (D) nursing satisfaction. Observation group (Intervention): underwent continuity of patient care interventions; Control group: no continuity of patient care interventions.

Three RCTs (Elkjaer et al., 2010; Ma et al., 2019) involving 357 IBD patients examined the disease recurrence rate, with Fig. 7A presenting a forest plot of the results, where in the random-effects model illustrated that continuity of patient care did not significantly reduce the disease recurrence rate in IBD patients (SMD = 0.94, 95% CI [0.53–1.67], *p* = 0.83; Fig. 7A).

#### Medication adherence rate

Four RCTs (Elkjaer et al., 2010; Chen et al., 2016; Wu, Fu & Kang, 2018) involving 433 IBD patients reported on medication adherence. Compared to conventional care, continuity of patient care significantly improved adherence to medication (RR = 1.59, 95% CI [1.21–2.10], *p* = 0.0009; Fig. 7B).

#### Medical advice compliance

Two RCTs (Elkjaer et al., 2010) involving 265 IBD patients reported on the medical advice compliance rate. Compared to the conventional care group, continuity of patient care significantly improved adherence to doctor orders (RR = 2.68, 95% CI [1.72–4.18], *p* < 0.0001; Fig. 7C).

#### Nursing satisfaction

Two RCTs (Chen et al., 2016; Sun et al., 2021) involving 162 IBD patients examined nursing satisfaction, and the results of the meta-analysis showed that continuity of patient care did not significantly improve nursing satisfaction in IBD patients (SMD = 1.19, 95% CI [0.97–1.45], *p* = 0.10; Fig. 7D).

### Publication bias

Publication bias was not assessed, given that less than ten studies were included in each outcome. A *p* value less than 0.05 was considered statistically significant.

## Discussion

This meta-analysis showed that continuity of patient care significantly improved quality of life and each dimension of quality of life from multiple angles (All *p* < 0.05), including bowel symptoms, systemic symptoms, emotional function, social functioning, SF-36. Nevertheless, significant heterogeneity was noted in the baseline characteristics and interventions of RCTs. However, the results remained unchanged after the heterogeneity test. This may be ascribed to continuity of patient care being easier for IBD patients to accept and adhere to due to their continuous care, safety, and reliability.

The impact of continuity of patient care on improving the QoL of IBD patients remains controversial. Quality of life is regarded as one of the most fundamental factors in evaluating the clinical efficacy of chronic disease management. Caregivers are generally able to accurately rate the QoL for IBD patients, timely explain self-care skills after discharge, and offer health guidance, psychological support, and social support. Telemedicine, as the most popular method of continuity of patient care, possesses unique advantages compared to other interventions. Application (APP), as a common vector of telemedicine, offers a useful adjunct for IBD patients in disease management (Yin et al., 2019). Specifically, it offers convenience for patients residing far from health providers, reduces the costs and risks of traveling long distances to hospitals, and fulfills the health needs of patients at any time as long as they have access to a mobile phone or computer (De Jong et al., 2020). During the COVID-19 pandemic, staying at home and avoiding crowds was crucial for IBD patients to prevent infection and maintain their health.

Noteworthily, patients have recognized the benefits of telemedicine, specially in providing insight into IBD conditions, monitoring in response to symptoms, and timely communication with healthcare providers. According to an earlier study, continuity of patient care did not improve the quality of life of IBD patients (Bilgrami et al., 2019). Ongoing research is investigating the causes underlying telemedicine not affecting the quality of life of patients (Quinn et al., 2019). A possible reason for this phenomenon is the decreased compliance of patients over time. Meanwhile, the safety of IBD patients undergoing continuity of patient care based on telemedicine platform management is a concern. The principal safety concern is design flaws in platform management. Engaging medical staff in reporting challenges associated with telemedicine platforms, involving patients in their own care, and use of interactive tools such as electronic medical records and personal health records may address these issues (Rubin et al., 2020; Sultan et al., 2020). Therefore, there is an urgent need to offer more diverse and personalized systems for these patients. By designing such telemedical methods, these patients might feel more comfortable managing their disease and enjoying a better quality of life (Flamant & Roblin, 2018).

Furthermore, previous studies reported that up to 30.5% of Crohn’s disease patients developed anxiety or depression (Häuser et al., 2011; Goodhand et al., 2012). In the event of psychological or psychiatric problems, continuity of patient care could timely assist in diagnosis and intervention. This study also yielded similar conclusions: continuity of patient care significantly alleviated the anxiety and depression of IBD patients (SMD = −1.98, 95% CI [−2.40 to −1.55], *p* < 0.00001; SMD = −1.82, 95% CI [−2.67 to −0.98], *p* < 0.0001). According to previous studies, continuity of patient care can aid in medication adherence and medical satisfaction (Atreja, 2018; Bethishou et al., 2019). Likewise, our study observed that medication adherence and doctor order compliance rates were higher in the continuity of patient care group (RR = 1.59, 95% CI [1.21–2.10], *p* = 0.0009) and (RR = 2.68, 95% CI [1.72–4.18], *p* < 0.0001) compared with conventional care. On the other hand, continuity of patient care did not significantly enhance patient satisfaction (SMD = 1.19, 95% CI [0.97–1.45], *p* = 0.10). Similar conclusions were drawn from some other reviews. Mi et al. (2024) performed a meta-analysis to explore the effects of community nursing for stroke survivors. They found that community nursing combined with routine nursing might relieve anxiety and depression, and improve daily activities and motor function. Han et al. (2025) also performed a meta-analysis to explore the effects of nurse-led interventions in colorectal cancer management throughout the cancer care continuum. They found that it could reduce stoma-related complications, relieve anxiety and depression, improve screening rate for colorectal cancer and quality of life. Overall, most of systematic meta-analysis confirmed the positive effects of continuity of patient care.

The studies in our analysis are the most precise and comprehensive, due to the largest sample size and the meta-analysis method. However, it is paramount to note that continuity of patient care may differ across studies, potentially introducing bias. Secondly, our selection of articles was limited to Chinese and English sources, potentially overlooking articles in other languages and introducing linguistic bias. Secondly, the reported duration of the training varied from 2 months to 12 months, which could have an impact on outcomes. Thirdly, a considerable proportion of the included studies lacked detailed reporting on key methodological domains (*e.g.*, randomisation, allocation concealment, and blinding). Consequently, the certainty of the evidence is limited, as we were unable to fully ascertain the internal validity of these trials. This highlights the need for future primary studies to adhere to the CONSORT statement to improve reporting quality. In addition, restricting to indexed journals may have introduced selection bias, and that the results should be interpreted with this in mind. Finally, due to the limited number of outcomes, subgroup analyses could not be performed. In the future, researchers can explore the subjective perceptions of patients using Cohen’s hermeneutic phenomenological approach (Bosco et al., 2025). Further prospective multicenter studies with large sample, qualitative research approaches, and longer follow-up periods in study to confirm our results are warranted.

## Conclusions

Overall, our findings demonstrated that continuity of patient care might improve the quality of life and concomitantly alleviate anxiety and depression in patients with inflammatory bowel disease compared to conventional care. However, continuity of patient care did not significantly reduce the recurrence rate for inflammatory bowel disease. This meta-analysis provided a new perspective for continuity of patient care in the field of inflammatory bowel disease. Healthcare workers could carry out continuity of patient care for patients with inflammatory bowel disease. Notwithstanding, due to large heterogeneity and non-robust effects, the results should be interpreted with caution.

##  Supplemental Information

10.7717/peerj.21429/supp-1 Supplemental Information 1 Search strategy of Cochrane and web of science

10.7717/peerj.21429/supp-2 Supplemental Information 2 Audience

10.7717/peerj.21429/supp-3 Supplemental Information 3 PRISMA checklist

## Additional Information and Declarations

### Data Availability

The following information was supplied regarding data availability: This is a PeerJ systematic review/meta-analysis.
