# Supplementary material for: Effect of continuity of patient care on quality of life and psychological state in patients with inflammatory bowel disease: a systematic review and meta-analysis of randomized controlled trials
Source: PeerJ. 2026 Jul 14;14:e21429. doi: 10.7717/peerj.21429 (PMC13378465; doi:10.7717/peerj.21429)
Supplement: Supplemental Information 1 [file peerj-14-21429-s001.docx]

**Search strategy of Cochrane**

#1 ("Inflammatory Bowel Diseases"):ti,ab,kw OR ("Inflammatory Bowel Disease"):ti,ab,kw OR ("Bowel Diseases, Inflammatory"):ti,ab,kw OR ("Crohn Disease"):ti,ab,kw OR ("Crohn's Enteritis"):ti,ab,kw OR ("Regional Enteritis"):ti,ab,kw OR ("Crohn's Disease"):ti,ab,kw OR ("Crohns Disease"):ti,ab,kw OR ("Inflammatory Bowel Disease 1"):ti,ab,kw OR ("Enteritis, Granulomatous"):ti,ab,kw OR ("Granulomatous Enteritis"):ti,ab,kw OR ("Enteritis, Regional"):ti,ab,kw OR ("Ileocolitis"):ti,ab,kw OR ("Colitis, Granulomatous"):ti,ab,kw OR ("Granulomatous Colitis"):ti,ab,kw OR ("Ileitis, Terminal"):ti,ab,kw OR ("Terminal Ileitis"):ti,ab,kw OR ("Ileitis, Regional"):ti,ab,kw OR ("Regional Ileitides"):ti,ab,kw OR ("Regional Ileitis"):ti,ab,kw OR ("Colitis, Ulcerative"):ti,ab,kw OR ("Idiopathic Proctocolitis"):ti,ab,kw OR ("Ulcerative Colitis"):ti,ab,kw OR ("Colitis Gravis"):ti,ab,kw OR ("Inflammatory Bowel Disease, Ulcerative Colitis Type"):ti,ab,kw

#2 ("Continuity of Patient Care"):ti,ab,kw OR ("Care Continuity, Patient"):ti,ab,kw OR ("Patient Care Continuity"):ti,ab,kw OR ("Continuum of Care"):ti,ab,kw OR ("Care Continuum"):ti,ab,kw OR ("Continuity of Care"):ti,ab,kw OR ("Care Continuity"):ti,ab,kw

#3 #1 and #2

**Search strategy of Web of Science**

1: (((((((((((((((((((((((((((TS=(Inflammatory Bowel Diseases)) OR TS=(Crohn Disease)) OR TS=(Colitis, Ulcerative)) OR AB=(Inflammatory Bowel Diseases)) OR AB=(Inflammatory Bowel Disease)) OR AB=(Inflammatory Bowel Disease)) OR AB=(Crohn Disease)) OR AB=(Crohn's Enteritis)) OR AB=(Regional Enteritis)) OR AB=(Crohn's Disease)) OR AB=(Crohns Disease)) OR AB=(Inflammatory Bowel Disease 1)) OR AB=(Enteritis, Granulomatous)) OR AB=(Granulomatous Enteritis)) OR AB=(Enteritis, Regional)) OR AB=(Ileocolitis)) OR AB=(Colitis, Granulomatous)) OR AB=(Granulomatous Colitis)) OR AB=(Ileitis, Terminal)) OR AB=(Terminal Ileitis)) OR AB=(Ileitis, Regional)) OR AB=(Regional Ileitides)) OR AB=(Regional Ileitis)) OR AB=(Colitis, Ulcerative)) OR AB=(Idiopathic Proctocolitis)) OR AB=(Ulcerative Colitis)) OR AB=(Colitis Gravis)) OR AB=(Inflammatory Bowel Disease, Ulcerative Colitis Type)

2: (((((((TS=(Continuity of Patient Care)) OR AB=(Continuity of Patient Care)) OR AB=(Care Continuity, Patient)) OR AB=(Patient Care Continuity)) OR AB=(Continuum of Care)) OR AB=(Care Continuum)) OR AB=(Continuity of Care)) OR AB=(Care Continuity)

3: #2 AND #1

4: (((AB=(randomized)) OR AB=(randomly)) OR AB=(trial)) OR AB=(groups)

5: #3 AND #4
