# Supplementary material for: Effect of continuity of patient care on quality of life and psychological state in patients with inflammatory bowel disease: a systematic review and meta-analysis of randomized controlled trials
Source: PeerJ. 2026 Jul 14;14:e21429. doi: 10.7717/peerj.21429 (PMC13378465; doi:10.7717/peerj.21429)
Supplement: Supplemental Information 2 [file peerj-14-21429-s002.docx]

The audience

Digestive physicians; general surgeons; general practitioners; nurses
